# RNA-Binding Proteins in Ageing and Age-Related Disease

**DOI:** 10.3390/neurolint18060112

**Published:** 2026-06-07

**Authors:** João Miguel Alves Ferreira, Sergii Tukaiev, Vaitsa Giannouli

**Affiliations:** 1 Institute of Pharmacology and Experimental Therapeutics, Faculty of Medicine, University of Coimbra, 3004-531 Coimbra, Portugal; 2 Coimbra Institute for Clinical and Biomedical Research (iCBR), Faculty of Medicine, University of Coimbra, 3004-531 Coimbra, Portugal; 3 Center for Innovative Biomedicine and Bio-Technology (CIBB), University of Coimbra, 3004-531 Coimbra, Portugal; 4 Faculty of Communication, Culture, and Society, Institute of Public Health, Università Della Svizzera Italiana, 6900 Lugano, Switzerland; 5 Higher Institute of Science Education and Technology, Taras Shevchenko National University of Kyiv, 01033 Kyiv, Ukraine; 6 Department of Psychology, Democritus University of Thrace, 68300 Didymoteicho, Greece

**Keywords:** RNA-binding proteins, ageing, cellular senescence, alternative splicing, TDP-43, neurodegeneration, post-transcriptional regulation

## Abstract

RNA-binding proteins (RBPs) are essential regulators of all aspects of RNA metabolism, including splicing, stability, localisation, translation, and degradation. Through their ability to recognise specific cis-elements in target transcripts, often via RNA-recognition motifs or other conserved domains, RBPs enable rapid cellular adaptation to stress and maintain proteostasis, particularly in post-mitotic tissues with limited transcriptional flexibility. Accumulating evidence positions RBPs as both modulators and drivers of the molecular hallmarks of ageing, including genomic instability, loss of proteostasis, mitochondrial dysfunction, cellular senescence, and chronic inflammation. This review synthesises peer-reviewed studies on the multifaceted roles of RNA-binding proteins in organismal ageing and age-related diseases. Key themes include the tissue- and age-dependent changes in expression of turnover and translation regulatory RBPs such as HuR (ELAVL1), AUF1 (HNRNPD), TIA-1, and tristetraprolin (ZFP36), which alter the stability of mRNAs encoding cell-cycle regulators, pro-inflammatory cytokines, and stress-response proteins. Systematic downregulation of core splicing factors, including PTBP1 and several heterogeneous nuclear ribonucleoproteins, drives widespread senescence-associated splicing alterations in pathways governing cell division, autophagy, DNA repair, and mitochondrial function, suggesting a causal contribution to the senescent phenotype. Prion-like RBPs such as TDP-43 and FUS exhibit age-dependent mislocalisation, nuclear depletion, and cytoplasmic aggregation, contributing to splicing defects, impaired RNA transport, and neurodegeneration in amyotrophic lateral sclerosis, frontotemporal dementia, and limbic-predominant age-related TDP-43 encephalopathy. Interactions between RBPs and non-coding RNAs, together with disrupted liquid–liquid phase separation dynamics, further exacerbate age-related decline. By integrating mechanistic studies from cellular and animal models with observations in human cohorts, this review underscores RBPs as central nodes linking multiple ageing hallmarks and highlights their potential as biomarkers and therapeutic targets to promote healthy ageing. Limitations of current models and priorities for future translational research are discussed.

## 1. Introduction

The biology of ageing has shifted from a passive view of accumulated stochastic damage to an active, modifiable process governed by interconnected molecular hallmarks that can be experimentally targeted to extend healthspan [1]. In their updated framework, López-Otín et al. [1] expanded the original nine hallmarks to twelve, explicitly incorporating disabled macroautophagy, chronic inflammation, and dysbiosis alongside classical features such as genomic instability, telomere attrition, epigenetic alterations, loss of proteostasis, deregulated nutrient sensing, mitochondrial dysfunction, cellular senescence, stem cell exhaustion, and altered intercellular communication. Within this expanded universe, post-transcriptional regulation of gene expression has gained prominence as both a downstream effector of these hallmarks and an active driver that amplifies their impact across tissues and over time [2].

RNA-binding proteins (RBPs) constitute a large and heterogeneous class of approximately 1500–2000 proteins in humans that interact with RNA through a variety of RNA-binding domains and intrinsically disordered regions [3,4]. These proteins control every stage of the RNA life cycle, from co-transcriptional splicing and polyadenylation in the nucleus to cytoplasmic localisation, translation, stability, and degradation [5]. Because post-transcriptional control allows cells to fine-tune protein production rapidly and locally without requiring new transcription, RBPs are particularly critical in long-lived post-mitotic cells such as neurons and cardiomyocytes, which have limited capacity for transcriptional reprogramming as they age [6]. Consequently, age-related changes in RBP expression, localisation, post-translational modification, or aggregation have profound consequences for cellular homeostasis and organismal decline.

Early recognition of the importance of RBPs in ageing came from coordinated efforts in the early 2010s. Gorospe [7] highlighted how RBPs regulate mRNA turnover and translation of transcripts encoding proteins central to age-related phenotypes, including muscle wasting, liver dysfunction, and neurodegeneration. This foundational perspective was built upon by systematic surveys of RBP expression across human tissues and during replicative senescence. Masuda et al. [8] demonstrated that turnover and translation regulatory RBPs (TTR-RBPs) such as HuR (ELAVL1), AUF1 (HNRNPD), TIA-1, and tristetraprolin (ZFP36/TTP) exhibit highly tissue-specific and age-dependent expression patterns. In most tissues, HuR, AUF1, and TIA-1 levels declined with donor age while TTP increased, producing corresponding shifts in the stability and translation of mRNAs bearing AU-rich elements in their 3′ untranslated regions. These mRNAs encode critical regulators of cell-cycle progression (p21, p16), inflammatory cytokines, and antioxidant enzymes, directly linking RBP dysregulation to the pro-inflammatory state and loss of proliferative capacity observed in aged tissues [8].

A parallel line of investigation has focused on pre-mRNA splicing, a process regulated by two major families of RBPs: heterogeneous nuclear ribonucleoproteins (hnRNPs) that often repress exon inclusion and serine/arginine-rich (SR) proteins that generally promote it. Splicing fidelity declines markedly with age, resulting in increased intron retention, exon skipping, and production of aberrant isoforms that can trigger nonsense-mediated decay or generate toxic proteins [9]. Dong et al. [10] performed a comprehensive transcriptome-wide analysis across multiple models of cellular senescence and identified consistent downregulation of a core subset of splicing regulatory RBPs, most notably PTBP1 and hnRNPUL1. This downregulation was accompanied by more than 400 senescence-associated differential splicing events enriched in functional pathways including cell division, DNA repair, autophagy, mitochondrial respiration, and proton transport. Importantly, experimental depletion of several of these RBPs was sufficient to induce senescence-like phenotypes, suggesting that loss of splicing factor expression may be causal rather than simply correlative [2,10].

Particularly compelling evidence for RBPs as drivers of age-related pathology comes from the study of aggregation-prone RBPs containing prion-like low-complexity domains. TDP-43 (encoded by TARDBP) and FUS are perhaps the best-characterised examples. Neumann et al. [11] first identified TDP-43 as the major component of ubiquitinated inclusions in amyotrophic lateral sclerosis (ALS) and frontotemporal lobar degeneration. Subsequent work established that nuclear depletion of TDP-43 leads to widespread splicing defects, particularly in long neuronal introns and cryptic exons, while cytoplasmic aggregates sequester other RBPs and disrupt mRNA transport and local translation at synapses [12]. The recognition of limbic-predominant age-related TDP-43 encephalopathy (LATE) as a distinct neuropathological entity that predominantly affects individuals over 80 years of age further underscores the age-dependent nature of TDP-43 proteinopathy [13,14]. Similar mechanisms operate for FUS and other hnRNPs with prion-like domains, whose phase-separation behaviour becomes pathological when proteostasis capacity declines with age [6].

These observations illustrate how RBPs sit at the intersection of multiple ageing hallmarks. Loss of proteostasis promotes RBP aggregation, which in turn exacerbates genomic instability through defective DNA repair protein expression, mitochondrial dysfunction through altered splicing of mitochondrial transcripts, and chronic inflammation through dysregulated SASP factor production [1,2]. Cellular senescence itself is heavily regulated post-transcriptionally. The senescence-associated secretory phenotype (SASP) comprises dozens of pro-inflammatory cytokines, chemokines, proteases, and growth factors whose mRNAs are stabilised or destabilised by RBPs such as HuR and TTP. With advancing age, the balance shifts toward stabilisation of SASP transcripts, propagating senescence to neighbouring cells and fuelling inflammaging [2].

Interactions between RBPs and non-coding RNAs add further complexity. Many long non-coding RNAs and circular RNAs that accumulate with age function as molecular sponges or scaffolds that modulate RBP availability and localisation [15]. Conversely, RBPs regulate microRNA biogenesis and activity, creating extensive regulatory networks that become rewired during ageing and in age-related diseases ranging from cardiovascular disease and sarcopenia to metabolic syndrome and cancer.

The present review provides an extensive, critical synthesis of the peer-reviewed literature on RBPs in ageing and age-related disease. It draws exclusively upon high-impact papers identified through systematic database searches, prioritising work published in the past two decades while incorporating foundational studies. All citations follow APA 7th edition author-date style.

By establishing RBPs as central integrative nodes that both respond to and amplify the molecular hallmarks of ageing, this review aims to provide a robust foundation for geroscience investigators and clinicians interested in targeting RNA metabolism to mitigate age-related functional decline and extend healthy lifespan [1,2].

Rather than focusing exclusively on individual RBPs or single disease entities, this review adopts an integrative lifespan-oriented perspective that critically examines how RNA-binding proteins interact with multiple hallmarks of ageing simultaneously, contribute to tissue-specific pathology, and potentially function as systems-level regulators of RNA homeostasis across ageing organisms.

### Methodological Approach

This narrative review was conducted through a structured literature search aimed at identifying key studies on RNA-binding proteins in ageing and age-related diseases. Searches were performed in PubMed, Scopus, and Web of Science databases using combinations of keywords including “RNA-binding proteins”, “ageing”, “cellular senescence”, “alternative splicing”, and “neurodegeneration”.

Priority was given to peer-reviewed articles published between 2000 and 2025, with emphasis on high-impact journals and seminal studies. Both experimental and review articles were included to ensure comprehensive coverage of mechanistic insights and translational relevance. Additional references were identified through citation tracking of key publications.

Only articles published in English were considered. Studies were selected based on relevance to the role of RBPs in the hallmarks of ageing, cellular senescence, and age-related diseases.

## 2. RBPs at the Intersection of the Hallmarks of Ageing

The twelve hallmarks of ageing articulated by López-Otín et al. [1] do not operate in isolation; rather, they form an interconnected network in which RBPs frequently serve as both sensors and effectors. Genomic instability, for example, activates DNA-damage responses that alter RBP phosphorylation and localisation. The p38 MAPK and JNK pathways, which are chronically activated in aged tissues, phosphorylate HuR, TDP-43, and several hnRNPs, changing their RNA-binding affinity and subcellular distribution [16]. This phosphorylation can promote cytoplasmic translocation of HuR, stabilising mRNAs encoding pro-inflammatory and pro-senescence factors while depleting nuclear splicing factors, thereby linking DNA damage to both senescence and splicing dysregulation [10].

Macroautophagy is essential for the degradation of persistent RBP aggregates that escape proteasomal turnover, thereby preserving cellular proteostasis under age-associated stress conditions. Experimental evidence demonstrates that autophagic flux is required for the clearance of pathological RNA-binding protein assemblies, including TDP-43-positive inclusions and stress granule-associated condensates, while age-related decline in lysosomal function promotes aggregate persistence and proteotoxicity [17,18,19]. This decline contributes directly to the stabilisation of aberrant phase-separated structures and enhances their transition into irreversible pathogenic assemblies. TDP-43, FUS, and hnRNPA1 contain low-complexity domains that drive liquid–liquid phase separation under normal conditions but readily convert to stable amyloid-like fibrils when autophagy is compromised, a state that becomes more prevalent with age [6]. The resulting aggregates not only cause gain-of-toxic-function effects but also induce loss-of-nuclear-function by sequestering these RBPs in the cytoplasm, leading to splicing errors in hundreds of transcripts critical for neuronal and muscular homeostasis [12,13].

Mitochondrial dysfunction and deregulated nutrient sensing further intersect with RBP biology. Many nuclear-encoded mitochondrial mRNAs are regulated by RBPs such as LRPPRC, SLIRP, and PUF proteins [20,21]. Age-related decline in these RBPs contributes to impaired oxidative phosphorylation and increased reactive oxygen species production, which in turn oxidise other RBPs, altering their function [20,21]. Nutrient-sensing pathways (mTOR, AMPK, insulin/IGF-1) modulate RBP activity through phosphorylation and acetylation. For instance, mTORC1 inhibition extends lifespan in part by altering translation of specific mRNA subsets controlled by RBPs, while chronic mTOR activation in aged cells promotes SASP translation through selective engagement of HuR and other translational RBPs [2].

Cellular senescence occupies a particularly central position. As noted earlier, Dong et al. [10] identified a core senescence-associated splicing signature driven by downregulation of PTBP1, hnRNPUL1, and related factors. Varesi et al. [2] propose that RBPs function as “common linkers” across age-related diseases precisely because they coordinate the senescent phenotype. The SASP is exquisitely regulated post-transcriptionally: HuR stabilises many SASP mRNAs, whereas TTP and AUF1 promote their decay. With replicative age or exposure to chronic stress, HuR activity predominates, amplifying sterile inflammation that drives tissue dysfunction [8]. Senescent cells also exhibit altered phase-separation dynamics, with persistent stress granules that sequester RBPs and impair normal RNA metabolism, creating a self-reinforcing loop. Persistent stress granules may progressively transition from dynamic liquid condensates into pathological insoluble aggregates, particularly under conditions of impaired proteostasis and chronic cellular stress [19,22,23].

Stem cell exhaustion is similarly influenced. RBPs regulate the balance between self-renewal and differentiation transcripts in haematopoietic, mesenchymal, and neural stem cells. Age-related changes in RBP expression bias splicing toward isoforms that favour differentiation or senescence at the expense of self-renewal, contributing to the well-documented decline in regenerative capacity [2,24].

Altered intercellular communication and chronic inflammation (“inflammaging”) are amplified by RBP-controlled SASP and by extracellular vesicles that carry specific RBPs and their bound RNAs between cells. Circulating RBPs and RBP–RNA complexes have been detected in the plasma of older individuals and correlate with frailty indices, suggesting both mechanistic and biomarker relevance [15].

Dysbiosis, the final hallmark in the López-Otín et al. [1] framework, also interfaces with RBPs. Gut microbiome-derived metabolites influence host RBP expression and phase separation in intestinal epithelial cells, while systemic inflammation driven by microbial products alters neuronal and hepatic RBP function, closing the circle between microbiome, inflammation, and RNA metabolism.

In summary, RBPs do not merely respond passively to the activation of individual hallmarks; they integrate signals across the entire network, often converting transient stresses into persistent maladaptive states. This central positioning makes them particularly attractive targets for gerotherapeutic interventions.

**Figure 1:**
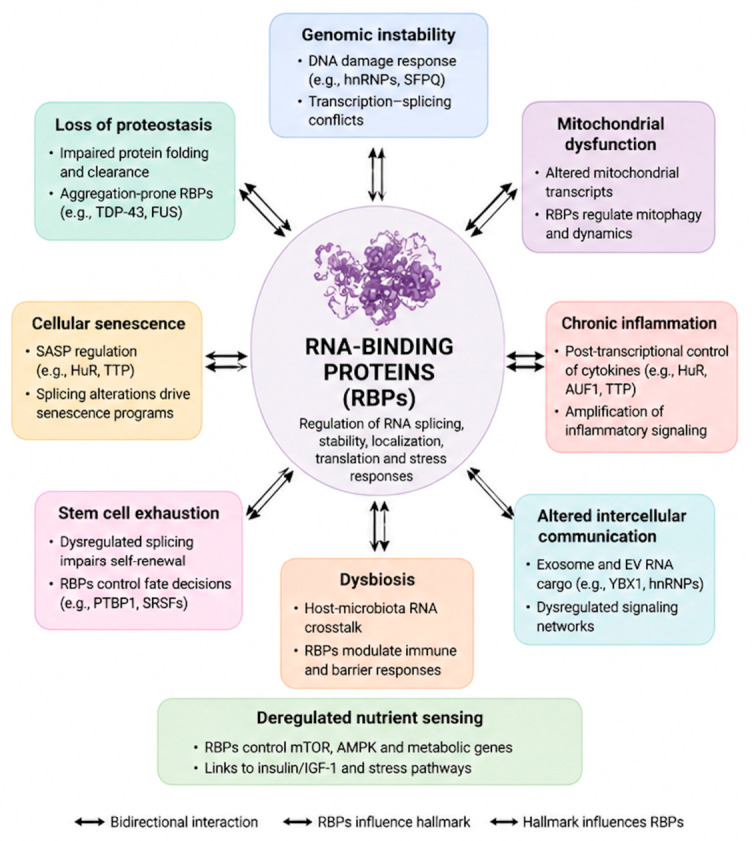
RNA-Binding Proteins as Integrative Regulators of the Hallmarks of Ageing. Note: Schematic representation of how RBPs interact bidirectionally with the twelve hallmarks of ageing, including genomic instability, proteostasis loss, mitochondrial dysfunction, and cellular senescence. RBPs regulate RNA metabolism at multiple levels, thereby acting as central nodes that integrate stress responses and propagate age-associated phenotypes. Created by the authors on 27 May 2026. Conceptual framework based on [1,2].

Given the extensive interconnections between RNA-binding proteins (RBPs) and the molecular hallmarks of ageing, a conceptual integrative framework is useful to visualise how disturbances in RNA metabolism propagate across multiple ageing-associated pathways simultaneously. Figure 1 summarises the central role of RBPs as bidirectional regulators and downstream effectors of biological ageing processes.

### RNA-Binding Proteins Across the Lifespan: From Development to Ageing

RNA-binding proteins (RBPs) implicated in ageing also play essential roles during embryonic development, tissue maturation, stem cell maintenance, and cellular differentiation. This developmental perspective is critical, as it supports the concept of antagonistic pleiotropy, whereby molecular functions that are beneficial during early life and reproductive fitness may become maladaptive in later life [25].

RBPs such as PTBP1, HuR (ELAVL1), FUS, TDP-43, and members of the heterogeneous nuclear ribonucleoprotein (hnRNP) family are highly expressed during embryogenesis and regulate lineage specification, neuronal differentiation, RNA localisation, synaptic maturation, and tissue morphogenesis [26,27]. PTBP1, for example, represses neuronal exon inclusion during early neurodevelopment and must subsequently decline to permit PTBP2-dependent neuronal maturation programmes [26]. Similarly, ELAVL family proteins regulate developmental transitions in mRNA stability and local translation in differentiating neurons.

Several age-related pathologies may therefore reflect partial reactivation, exhaustion, or dysregulation of developmental RNA-processing programmes. Aberrant inclusion of fetal splice isoforms has been documented in Alzheimer’s disease, amyotrophic lateral sclerosis, and frontotemporal dementia, suggesting that ageing tissues may progressively lose splicing fidelity and revert toward immature transcriptomic states [28]. Senescent cells likewise exhibit transcriptomic features resembling incomplete developmental reprogramming, including altered splicing fidelity and dysregulated stress-granule dynamics.

This lifespan perspective also helps explain the pronounced tissue specificity of RBP dysfunction during ageing. Post-mitotic tissues such as the brain and skeletal muscle remain highly dependent on tightly regulated RNA-processing networks throughout life and possess limited regenerative capacity, rendering them particularly vulnerable to RBP dysregulation.

Overall, integrating developmental biology with geroscience provides a more comprehensive framework for understanding how RBPs contribute both to organismal maturation and to late-life pathological decline.

## 3. The Central Role of RNA-Binding Proteins in Cellular Senescence and the Senescence-Associated Secretory Phenotype

Cellular senescence represents a state of stable proliferative arrest that cells enter in response to various stressors, including telomere shortening, persistent DNA damage, oncogene activation, mitochondrial dysfunction, and oxidative stress [1]. Although senescence functions as a tumour-suppressive mechanism in young organisms by preventing the propagation of damaged cells, its accumulation in aged tissues contributes significantly to organismal decline, chronic sterile inflammation (inflammaging), impaired tissue regeneration, and the progression of multiple age-related diseases [2]. A defining feature of senescent cells is the acquisition of the senescence-associated secretory phenotype (SASP), a highly heterogeneous secretome enriched in pro-inflammatory cytokines (for example, IL-6 and IL-8), chemokines (CXCL1, CXCL8), matrix metalloproteinases (MMP-1, MMP-3), growth factors, and extracellular matrix remodelling proteins. The SASP exerts both local and systemic effects by propagating senescence to neighbouring cells in a paracrine fashion, promoting chronic inflammation, altering the tissue microenvironment, and contributing to pathologies such as fibrosis, osteoarthritis, neurodegeneration, and cancer [2,29].

RNA-binding proteins (RBPs) have emerged as master regulators and potential common linkers of both the establishment and maintenance of the senescent state and the dynamic control of SASP composition and intensity. By governing every aspect of RNA metabolism (including alternative splicing, mRNA stability, nuclear export, cytoplasmic localisation, translation efficiency, and degradation), RBPs enable rapid, transcription-independent reprogramming of gene expression in response to senescence-inducing stimuli [2]. This post-transcriptional layer of control is particularly critical in non-dividing or slowly dividing cells, where transcriptional plasticity is limited. Consequently, age-dependent alterations in RBP expression, subcellular localisation, post-translational modification, phase-separation behaviour, or aggregation status can convert transient stress responses into persistent maladaptive phenotypes [10,29].

One of the most reproducible molecular hallmarks of senescence is a profound transcriptome-wide remodelling of alternative splicing patterns. Dong et al. [10] performed a systematic analysis across multiple independent models of human cellular senescence (replicative, oncogene-induced, therapy-induced, and DNA damage-induced) and identified a core signature of 406 senescence-associated differential splicing events. These events were strongly enriched in transcripts regulating cell-cycle progression, DNA repair pathways, autophagy, mitochondrial function, proton transport, and chromatin organisation. This splicing reprogramming was driven primarily by the consistent and coordinated downregulation of a subset of key splicing-regulatory RBPs, most notably PTBP1 (polypyrimidine tract-binding protein 1), hnRNPUL1, and several other heterogeneous nuclear ribonucleoproteins (hnRNPs). Many of these RBPs are themselves subject to alternative splicing during senescence, generating isoforms with altered function and establishing potential self-reinforcing feedback loops. Experimental knockdown of PTBP1 or hnRNPUL1 using RNA interference was sufficient to trigger multiple senescence hallmarks, including permanent cell-cycle arrest, increased senescence-associated β-galactosidase activity, activation of a partial SASP programme, and characteristic morphological changes [10]. These findings strongly support a causal rather than merely correlative role for splicing factor loss in senescence. Parallel reductions in the expression or activity of serine/arginine-rich (SR) proteins (such as SRSF1, SRSF3, and SRSF7) and additional hnRNP family members have been documented in fibroblasts from chronologically aged donors, in cells from patients with progeroid syndromes (including Hutchinson-Gilford progeria), and across multiple tissues in naturally aged mice and humans [2,29].

The functional consequences of these splicing alterations extend far beyond classical cell-cycle regulators such as p53, p16^INK4a, and p21^CIP1. Senescence-associated splicing switches frequently produce isoforms with modified stability, altered coding potential, or dominant-negative functions that reinforce proliferative arrest and SASP activation. For instance, changes in the splicing of lamin A transcripts generate progerin-like isoforms, while altered splicing of mitochondrial mRNAs impairs oxidative phosphorylation, elevates reactive oxygen species production, and feeds back into DNA damage signalling. RBPs such as hnRNPA1, hnRNPA2B1, and SRSF1 tightly regulate these events; their reduced nuclear abundance or altered phosphorylation status (often mediated by stress-activated kinases such as p38 MAPK, JNK, or CLK1, which display heightened activity in aged cells) contributes to the progressive loss of splicing fidelity that characterises both replicative and stress-induced senescence [2,10].

The lncRNA MALAT1, for example, sequesters SR proteins in nuclear speckles and modulates their phosphorylation and splicing activity, thereby influencing global alternative splicing programmes during ageing and stress responses [30].

In parallel with splicing regulation, turnover and translation regulatory RBPs (TTR-RBPs) exert precise control over SASP mRNA stability and translation. HuR (ELAVL1) is among the most extensively studied in this context. HuR typically binds AU-rich elements (AREs) in the 3′ untranslated regions of target transcripts and promotes their stability and translation. Numerous SASP components, including IL-6, IL-8, TNF-α, CXCL1, and several MMPs, are ARE-containing mRNAs whose half-lives increase dramatically in senescent cells through HuR activity. In proliferating cells, HuR is predominantly nuclear, but senescence-inducing stimuli trigger its cytoplasmic translocation via phosphorylation, methylation, or acetylation, enabling it to stabilise pro-inflammatory transcripts while simultaneously repressing certain pro-proliferative mRNAs [8,29]. This dual functionality allows a single RBP to coordinate both cell-cycle exit and the inflammatory secretory programme. Consistent with this model, HuR protein levels and cytoplasmic activity are elevated in multiple senescent cell types (fibroblasts, endothelial cells, epithelial cells, and astrocytes), and its targeted depletion or pharmacological inhibition attenuates key SASP factors without fully reversing proliferative arrest [2].

Opposing HuR are destabilising RBPs, most notably tristetraprolin (TTP/ZFP36) and AUF1 (HNRNPD). TTP recruits the CCR4-NOT deadenylase complex to accelerate the degradation of ARE-containing SASP transcripts. In young cells, high TTP activity maintains tight control over inflammatory cytokine production. During senescence and chronological ageing, however, although TTP expression may increase, its functional activity is often suppressed through phosphorylation by p38 MAPK or MK2, leading to its sequestration into inactive cytoplasmic complexes. This functional inactivation, combined with sustained HuR dominance, produces a net shift toward SASP mRNA stabilisation and chronic low-grade inflammation ([7,8], as cited in [2]). AUF1 exhibits isoform-specific and context-dependent roles, with certain isoforms promoting decay of p16^INK4a and p21 mRNAs in young cells; the age-associated decline in these isoforms contributes to the stabilisation of senescence effectors [8]. These reciprocal changes in TTR-RBP balance illustrate how RBPs translate acute stress signals into the persistent, self-sustaining inflammatory state characteristic of senescent cells.

Additional layers of regulation involve translational control, liquid–liquid phase separation, and interactions with non-coding RNAs. RBPs such as TIA-1, G3BP1, and FUS nucleate stress granules and processing bodies that sequester specific SASP transcripts in a translationally silent state during the early phases of senescence. In chronic senescence, however, these dynamics become pathological; persistent or aberrant phase-separated condensates can evolve into stable aggregates when proteostasis capacity declines, particularly in aged cells [2,29]. Senescent cells also exhibit increased expression of certain long non-coding RNAs (lncRNAs) and circular RNAs (circRNAs) that act as molecular sponges or scaffolds. The lncRNA MALAT1, for example, modulates SR protein phosphorylation and nuclear speckle organisation, thereby influencing global splicing patterns, while several age-associated circRNAs contain multiple binding sites for HuR, PTBP1, or hnRNPA1, effectively titrating these RBPs away from their canonical mRNA targets and further rewiring post-transcriptional networks ([15] as cited in [2]).

Prion-like RBPs such as TDP-43 and FUS also interface directly with senescence pathways. Nuclear depletion of TDP-43, frequently observed in aged neurons and glial cells, produces splicing defects that can trigger cell-autonomous senescence programmes, particularly in supporting glial cells, thereby contributing to neuroinflammation. Cytoplasmic TDP-43 aggregates activate the cGAS-STING innate immune pathway, reinforcing SASP activation in both cell-autonomous and paracrine manners [11,13]. Analogous mechanisms operate for FUS and certain hnRNPs containing low-complexity domains, whose phase-separation properties become dysregulated when autophagy and proteostasis decline with age [6].

In vivo evidence reinforces these cellular observations. In murine models of accelerated ageing (for example, Ercc1^Δ/−mice) and in naturally aged cohorts, downregulation of splicing-regulatory RBPs such as PTBP1 and multiple SRSF proteins correlates strongly with increased senescent cell burden in liver, kidney, skeletal muscle, and brain. Pharmacological or genetic senolytic clearance of senescent cells partially restores physiological RBP expression profiles, indicating bidirectional reinforcement between senescence and RBP dysregulation [2]. In human post-mortem tissues and biopsies from patients with age-related conditions (osteoarthritis, idiopathic pulmonary fibrosis, cardiovascular disease, and Alzheimer’s disease), altered localization or abundance of RBPs such as HuR, TDP-43, and PTBP1 is consistently observed within p16^INK4a-positive or SASP-positive cell clusters [29].

Notably, the relationship is not uniformly detrimental. Certain RBPs appear protective; stabilisation or overexpression of specific SR proteins or AUF1 isoforms can delay replicative senescence in cultured fibroblasts, while interventions known to extend healthspan (such as caloric restriction, mTOR inhibitors, or senolytics) partially normalise RBP levels, splicing fidelity, and SASP output [1,2]. These findings suggest that selective modulation of RBP function could suppress the deleterious inflammatory consequences of senescence while preserving its tumour-suppressive roles.

In conclusion, RBPs occupy a privileged integrative position at the core of cellular senescence. They orchestrate the splicing changes that establish and reinforce proliferative arrest, fine-tune the stability, localisation, and translation of SASP transcripts to control the magnitude and composition of the secretory programme, and mediate extensive crosstalk with other ageing hallmarks, including proteostasis collapse, mitochondrial dysfunction, genomic instability, and chronic inflammation. The coordinated downregulation of core splicing RBPs [10], the functional rebalancing of TTR-RBPs that favours SASP mRNA stabilisation [8], and the pathological phase transitions of aggregation-prone RBPs [2] collectively position this protein superfamily as both driver and effector of the senescent phenotype. As argued by Varesi et al. [2], RBPs may function as a molecular “common linker” across diverse age-related diseases precisely because of their central role in coordinating senescence and the SASP. A deeper mechanistic understanding of these processes is therefore essential for the development of next-generation gerotherapeutic strategies aimed at selectively targeting pathogenic aspects of senescence to extend human healthspan.

Collectively, these findings position RBPs as central regulators of cellular senescence and the senescence-associated secretory phenotype (SASP). Through coordinated control of mRNA stability, alternative splicing, stress responses, and inflammatory signalling, RBPs help determine whether senescent cells remain transiently adaptive or become chronically pathogenic during ageing.

## 4. Profiles of Key Individual RNA-Binding Proteins in Ageing and Age-Related Disease

### 4.1. HuR (ELAVL1)

HuR (human antigen R, encoded by ELAVL1) is a ubiquitously expressed member of the ELAV/Hu family of RBPs that primarily recognises AU-rich elements (AREs) in the 3′ untranslated regions of target mRNAs, generally promoting their stability and translation. In young cells, HuR is predominantly nuclear, where it participates in pre-mRNA processing, but it undergoes stimulus-dependent nucleocytoplasmic shuttling in response to stress, DNA damage, oxidative stress, or senescence-inducing signals [8]. Phosphorylation by p38 MAPK, JNK, or PKC, as well as methylation by CARM1, enhances its cytoplasmic translocation and RNA-binding affinity. In the cytoplasm, HuR stabilises numerous transcripts critical for stress responses, cell-cycle control, and inflammation [2].

With advancing age and in senescent cells, HuR exhibits increased cytoplasmic localisation and sustained activity toward pro-inflammatory and pro-senescence mRNAs. Masuda et al. [8] demonstrated in a comprehensive human tissue array that HuR levels generally decline in most organs with donor age, yet its cytoplasmic fraction and functional impact on specific targets increase in senescent fibroblasts, endothelial cells, and epithelial cells. This paradoxical shift enables HuR to stabilise SASP mRNAs encoding IL-6, IL-8, TNF-α, CXCL1, and several matrix metalloproteinases, thereby amplifying inflammaging. HuR also stabilises mRNAs encoding p21^CIP1 and p16^INK4a under certain conditions while repressing translation of certain proliferative transcripts, reinforcing proliferative arrest [29]. In age-related diseases, elevated cytoplasmic HuR contributes to chronic inflammation in osteoarthritis, atherosclerosis, and neurodegenerative conditions. In skeletal muscle, dysregulated HuR exacerbates sarcopenia by altering the stability of myogenic regulatory factors and inflammatory mediators [7]. Pharmacological inhibition of HuR–RNA interactions or its cytoplasmic translocation has shown promise in preclinical models for attenuating SASP-driven inflammation without completely ablating HuR’s protective stress-response functions [2]. Thus, HuR exemplifies an RBP whose beneficial adaptive role in youth becomes maladaptive when chronically activated in the aged cellular environment.

### 4.2. AUF1 (HNRNPD)

AUF1 (AU-rich element RNA-binding protein 1, also known as heterogeneous nuclear ribonucleoprotein D) exists in four isoforms generated by alternative splicing and exerts context-dependent effects on mRNA stability—some isoforms promote decay while others stabilise transcripts. AUF1 competes with HuR for ARE-binding sites and is a key antagonist of HuR-mediated stabilisation of SASP and senescence-associated transcripts [8]. In young tissues, specific AUF1 isoforms promote rapid turnover of mRNAs encoding p21, p16, and pro-inflammatory cytokines, thereby restraining senescence and inflammation.

During ageing, there is a progressive decline in functional AUF1 isoforms in multiple human tissues, correlating with donor age and replicative senescence in cultured cells [8]. This reduction allows HuR dominance, resulting in stabilisation of senescence effectors and SASP components. AUF1 also participates in DNA repair and telomere maintenance; its deficiency exacerbates genomic instability, linking it directly to another ageing hallmark [1]. In neurodegenerative disease models, altered AUF1 activity contributes to dysregulated expression of neuronal transcripts, while in liver and adipose tissue it influences metabolic homeostasis and lipid handling, processes that deteriorate with age. Restoration of specific AUF1 isoforms in senescent cell models delays senescence onset and reduces SASP output, highlighting its protective potential [2]. The reciprocal relationship between AUF1 and HuR illustrates how shifts in the balance of opposing TTR-RBPs can tip cells toward a chronic senescent and inflammatory state.

### 4.3. Tristetraprolin (TTP/ZFP36)

Tristetraprolin (TTP, encoded by ZFP36) is a CCCH zinc-finger RBP that binds AREs and recruits the CCR4–NOT deadenylase complex to promote rapid mRNA deadenylation and decay, particularly of pro-inflammatory transcripts. In youthful cells, TTP acts as a critical brake on inflammation by destabilising mRNAs encoding TNF-α, IL-6, IL-1β, and COX-2. Its activity is tightly regulated by phosphorylation; under acute stress, MAPK-activated protein kinase 2 (MK2) phosphorylates TTP, sequestering it in inactive complexes and permitting transient inflammatory responses [7].

In ageing and senescence, although total TTP protein levels may increase in some tissues, its functional activity is markedly impaired due to sustained inhibitory phosphorylation and altered localisation [8]. This functional inactivation removes the counterbalance to HuR, resulting in prolonged half-lives of SASP mRNAs and persistent inflammaging. In age-related diseases such as rheumatoid arthritis, cardiovascular disease, and metabolic syndrome, defective TTP activity contributes to unresolved chronic inflammation. In the brain, TTP dysregulation has been linked to neuroinflammation in Alzheimer’s disease models. Genetic or pharmacological strategies that enhance TTP expression or activity have shown anti-inflammatory and anti-senescence effects in preclinical ageing studies [2]. TTP therefore represents a promising node for therapeutic restoration to mitigate the deleterious secretory phenotype of senescent cells.

### 4.4. PTBP1 and Core Splicing Regulatory RBPs (hnRNPs and SR Proteins)

PTBP1 (polypyrimidine tract-binding protein 1) is a multifunctional hnRNP that typically represses exon inclusion by binding to intronic or exonic splicing silencers. It also regulates mRNA stability, localisation, and translation. Dong et al. [10] identified PTBP1 as one of the most consistently downregulated splicing factors across diverse senescence models, alongside hnRNPUL1, SRSF1, SRSF7, QKI, RBFOX2, HNRNPK, and HNRNPM. This coordinated downregulation drives a core set of over 400 senescence-associated splicing events affecting cell-cycle regulators, DNA repair factors, autophagy genes, and mitochondrial transcripts.

Reduced PTBP1 levels lead to inclusion of exons that introduce premature stop codons or produce pro-senescence protein isoforms, reinforcing cell-cycle arrest and mitochondrial dysfunction [10]. Similar declines in other hnRNP and SR proteins occur in progeroid syndromes, naturally aged human tissues, and murine ageing models [2]. These changes are not merely correlative; siRNA-mediated depletion of PTBP1 or SRSF1 is sufficient to induce senescence-like phenotypes. In age-related diseases, splicing dysregulation driven by these RBPs contributes to aberrant isoforms in neurodegeneration (e.g., tau splicing in Alzheimer’s disease), cardiomyopathy, and cancer. Therapeutic modulation of SRSF or hnRNP activity using small molecules or antisense oligonucleotides is an active area of translational research aimed at correcting age-associated splicing defects [29].

### 4.5. TDP-43 (TARDBP)

TAR DNA-binding protein 43 (TDP-43) is a nuclear RBP containing two RNA-recognition motifs and a prion-like low-complexity domain. It regulates alternative splicing, particularly of long neuronal introns, mRNA transport, and local translation at synapses. Neumann et al. [11] identified TDP-43 as the major protein component of ubiquitinated inclusions in amyotrophic lateral sclerosis (ALS) and frontotemporal lobar degeneration. With ageing, TDP-43 undergoes progressive nuclear depletion and cytoplasmic mislocalisation and aggregation, even in individuals without overt neurodegenerative disease. This pathology is now recognised as a defining feature of limbic-predominant age-related TDP-43 encephalopathy (LATE), which affects up to 25–50% of individuals over 80 years of age and contributes to cognitive decline independently of Alzheimer’s disease pathology [13].

Nuclear loss of TDP-43 produces widespread splicing defects, including inclusion of cryptic exons that trigger nonsense-mediated decay or toxic protein production. Nuclear depletion of TDP-43, consistently observed in ageing cortical and glial populations, induces cryptic exon inclusion, transcriptomic instability, and loss of RNA surveillance fidelity, thereby activating stress-responsive pathways capable of initiating senescence-like cellular programmes [12,31,32]. Cytoplasmic aggregates sequester other RBPs, impair RNA transport, and disrupt proteostasis, linking TDP-43 pathology to multiple ageing hallmarks [2,6]. In skeletal muscle, TDP-43 aggregates contribute to sarcopenia-like phenotypes. Therapeutic approaches targeting TDP-43 aggregation, nuclear import, or downstream splicing defects are under intense investigation.

### 4.6. FUS and Related Prion-like RBPs (hnRNPA1, hnRNPA2B1)

Fused in sarcoma (FUS) shares many properties with TDP-43, including RNA-recognition motifs, a prion-like domain, and roles in splicing, transcription, and DNA repair. Mutations in FUS cause familial ALS, while wild-type FUS aggregates in sporadic cases and aged brains. Age-dependent impairment of nucleocytoplasmic transport and declining proteostasis promote FUS cytoplasmic accumulation and aberrant phase separation into toxic condensates [6]. Similar mechanisms apply to hnRNPA1 and hnRNPA2B1, whose mutations cause multisystem proteinopathy and whose wild-type forms aggregate in aged tissues and stress granules that persist in senescence [2]. These proteins illustrate how ageing compromises the biophysical properties that normally enable dynamic, beneficial ribonucleoprotein granule formation.

### 4.7. Additional RBPs: TIA-1, G3BP1/G3BP2, SFPQ and YBX1

Recent primary evidence has further expanded the role of stress-granule-associated RBPs in ageing and neurodegeneration. Takayama et al. [33] demonstrated that the RNA-binding proteins PSF (SFPQ) and G3BP2 cooperate in the nucleus to regulate neuronal gene expression and maintain neuronal viability. Both proteins were significantly reduced in aged mouse brains and in human Alzheimer’s disease tissue, suggesting that age-dependent loss of RBP-mediated RNA regulation contributes directly to neuronal dysfunction and dementia. Importantly, the authors showed that PSF–G3BP2 interactions influence post-transcriptional mRNA stability and neuronal survival pathways, providing mechanistic evidence that alterations in RBP networks are not merely consequences of ageing but may actively drive neurodegenerative processes [2]. YBX1 (Y-box binding protein 1) regulates mRNA stability and translation of oxidative stress-response transcripts. In aged tissues and osteoarthritis cartilage, YBX1 shows reduced expression and altered cytoplasmic localization, impairing its protective function against cellular stress [2]. These RBPs further expand the network of post-transcriptional control that deteriorates during ageing. In summary, individual RBPs exhibit distinct yet interconnected age-related perturbations. TTR-RBPs such as HuR, AUF1, and TTP shift the balance of mRNA stability toward chronic inflammation [8], core splicing factors including PTBP1 and hnRNPs drive senescence-associated isoform switches [10], and prion-like RBPs such as TDP-43 and FUS undergo aggregation-linked loss- and gain-of-function pathologies [6,11,13]. As Varesi et al. [2] emphasise, these proteins function as common molecular linkers across age-related diseases through their coordinated regulation of senescence and the SASP. Understanding their individual and collective behaviours provides a foundation for the development of targeted biomarkers and therapeutics.

Although individual RBPs exhibit distinct molecular functions, several recurrent themes emerge across ageing biology, including altered mRNA stability, dysregulated alternative splicing, defective stress-granule dynamics, and impaired proteostasis. Table 1 summarises the principal RBPs discussed throughout this review, their major physiological roles, age-associated alterations, related diseases, and therapeutic implications.

Key takeaway: Distinct RNA-binding proteins exhibit specific age-associated dysfunctions, yet converge mechanistically through disruption of RNA surveillance, phase-separation homeostasis, and proteostatic regulation.

## 5. RBPs in Organ-Specific Age-Related Diseases

This section synthesises the evidence linking specific RBPs to the pathogenesis and progression of major age-related diseases, drawing upon mechanistic studies in cellular and animal models, human cohort data, and post-mortem analyses. Particular emphasis is placed on how the same RBP can exert protective functions in youth yet drive maladaptive processes in aged tissues, consistent with the principle of antagonistic pleiotropy [6,8].

### 5.1. Neurodegenerative Diseases

Neurodegenerative disorders represent the most extensively studied arena of RBP pathology in ageing. TDP-43 (TARDBP) and FUS are paradigmatic examples of RBPs whose prion-like low-complexity domains render them susceptible to age-related mislocalisation and aggregation. Neumann et al. [11] first demonstrated that TDP-43 constitutes the primary component of ubiquitinated inclusions in amyotrophic lateral sclerosis (ALS) and frontotemporal lobar degeneration (FTLD). In the vast majority of sporadic ALS cases and approximately 45% of FTLD cases, TDP-43 undergoes nuclear depletion accompanied by cytoplasmic hyperphosphorylation, ubiquitination, and aggregation. These events produce both loss-of-nuclear-function (leading to widespread splicing defects, particularly in long neuronal introns and cryptic exons) and cytoplasmic gain-of-toxic-function through sequestration of other RBPs and disruption of mRNA transport and local translation at synapses [6,12].

The recognition of limbic-predominant age-related TDP-43 encephalopathy (LATE) as a distinct entity has further highlighted the age-dependent nature of TDP-43 proteinopathy. LATE affects 20–50% of individuals over 80 years of age and contributes to amnestic cognitive decline that overlaps clinically with Alzheimer’s disease but shows distinct neuropathological features, predominantly involving the amygdala, hippocampus, and middle frontal gyrus [13]. Unlike familial ALS/FTD, LATE typically occurs without TARDBP mutations, suggesting that age-related decline in proteostasis, nucleocytoplasmic transport, and RNA metabolism creates a permissive environment for wild-type TDP-43 pathology [14]. Similar mechanisms operate for FUS, whose aggregation is observed in a subset of ALS/FTD cases and whose phase-separation properties become dysregulated with declining autophagy and rising oxidative stress in the ageing brain [2].

Recent evidence further implicates stress-granule-associated RBPs in Alzheimer’s disease pathology. Increasing evidence indicates that dysregulated stress granule assembly and altered RBP localisation contribute to neurodegenerative processes by disrupting RNA metabolism and cellular proteostasis [22,34]. Several stress-granule-associated RBPs also perform essential nuclear functions related to RNA processing, transcriptional regulation, and genomic stability, suggesting that their mislocalisation or functional depletion may compromise nuclear RNA homeostasis during disease progression. This disruption may promote transcriptomic instability, impair stress adaptation, and reinforce pathological protein aggregation, ultimately contributing to neurodegenerative decline and age-associated cellular dysfunction [2,34].

Beyond TDP-43 and FUS, other RBPs contribute to neurodegeneration. Altered expression or function of splicing factors such as PTBP1, hnRNPA1, and SRSFs leads to aberrant isoforms of tau, amyloid precursor protein, and synaptic transcripts in Alzheimer’s disease and Parkinson’s disease [10]. HuR stabilises mRNAs encoding neuroinflammatory mediators and oxidative stress-response proteins; its cytoplasmic accumulation in aged neurons exacerbates neuroinflammation and synaptic loss [29]. These changes intersect with mitochondrial dysfunction and impaired DNA repair, two prominent hallmarks of brain ageing [1]. Therapeutic strategies targeting TDP-43 nuclear import, aggregation propensity, or downstream splicing defects (including antisense oligonucleotides) are under active investigation and show promise in preclinical models of ALS, FTD, and LATE.

### 5.2. Musculoskeletal Ageing and Sarcopenia

Sarcopenia, the progressive loss of skeletal muscle mass, strength, and function with advancing age, is a major contributor to frailty and reduced quality of life in older adults. RBPs play prominent roles in muscle homeostasis, regeneration, and age-related decline. TDP-43 pathology extends beyond the nervous system to skeletal muscle. Aggregates of TDP-43 are characteristic of sporadic inclusion body myositis (sIBM), the most common inflammatory myopathy in individuals over 50 years of age, as well as in rimmed-vacuole myopathies, oculopharyngeal muscular dystrophy, and myofibrillar myopathies [35]. In these conditions, cytoplasmic TDP-43 inclusions are accompanied by altered splicing of sarcomeric transcripts (including TTN and NEB) and upregulation of muscle regeneration genes, suggesting both loss-of-function and toxic gain-of-function mechanisms [2,35].

HuR is equally critical in muscle. During myogenesis, HuR coordinates the stability and translation of transcripts encoding MyoD, myogenin, p21, and acetylcholine receptor subunits. In aged muscle, the balance between HuR and destabilising RBPs such as TTP and AUF1 shifts, favouring chronic inflammation and impaired myogenic differentiation [8]. HuR also regulates the stability of pro-inflammatory cytokine mRNAs, including TNF-α and IL-6, as well as enzymes such as nitric oxide synthase 2 (NOS2), processes that can contribute to inflammation-associated muscle dysfunction when dysregulated [36,37]. Conditional deletion or inhibition of HuR in preclinical models attenuates certain inflammatory aspects of muscle ageing while revealing its dual role in maintaining muscle differentiation programmes.

Core splicing factors downregulated in senescence, including PTBP1 and members of the hnRNP and SR families, impair the regenerative capacity of satellite cells and alter the splicing of transcripts essential for mitochondrial function and autophagy in myofibres [10]. These molecular changes translate into reduced muscle stem cell function, increased fibrosis, and sarcopenic phenotypes. TDP-43 and HuR therefore represent convergent nodes linking neurogenic and myogenic aspects of age-related motor decline, with therapeutic potential for oligonucleotide-based splicing correction or small-molecule modulation of RBP activity.

### 5.3. Cardiovascular Diseases

Ageing is the dominant risk factor for atherosclerosis, hypertension, heart failure with preserved ejection fraction, and vascular stiffness. RBPs regulate endothelial activation, vascular smooth muscle cell (VSMC) phenotype switching, macrophage foam cell formation, and cardiac fibrosis. HuR exhibits highly cell-specific effects in the vasculature. In endothelial cells, HuR regulates the stability of transcripts involved in inflammatory and immune responses, contributing to endothelial activation and monocyte recruitment [38]. In contrast, macrophage HuR promotes pro-inflammatory cytokine production and monocyte recruitment, while VSMC HuR appears largely atheroprotective by limiting excessive proliferation, inflammation, and plaque instability [38,39]. Splicing factors such as QKI, SRSFs, and hnRNPs are downregulated or mislocalised in aged vessels and atherosclerotic lesions, leading to aberrant isoforms of fibronectin, myosin heavy chain, and inflammatory mediators that promote plaque instability and vascular remodelling [2,10]. TDP-43 and FUS contribute to vascular inflammation and endothelial senescence through SASP amplification and impaired DNA repair. In the heart, HuR stabilises transcripts driving pathological hypertrophy and fibrosis, while altered splicing of titin and other sarcomeric genes, driven by reduced RBP fidelity, contributes to diastolic dysfunction in ageing myocardium [39].

These observations underscore the context-dependent nature of RBP function: the same protein (e.g., HuR) can be detrimental in endothelium and macrophages yet protective in VSMCs. Such complexity necessitates cell-specific or stage-specific therapeutic approaches, including targeted delivery of HuR inhibitors or splicing modulators to atherosclerotic plaques.

### 5.4. Metabolic Disorders, Hepatic Ageing, and Related Conditions

Age-related metabolic dysfunction, non-alcoholic fatty liver disease (NAFLD), type 2 diabetes, and obesity are tightly linked to RBP dysregulation. Hepatic HuR regulates mRNAs involved in lipid metabolism and inflammatory pathways, and its dysregulation may contribute to altered lipid homeostasis and hepatic inflammation [38]. AUF1 and TTP modulate the stability of transcripts involved in insulin signalling, gluconeogenesis, and lipid metabolism; their age-related decline promotes insulin resistance and hepatic steatosis [2,8].

In adipose tissue, senescence of preadipocytes driven by reduced PTBP1 and hnRNP activity leads to impaired adipogenesis, ectopic lipid deposition, and systemic inflammation [10]. TDP-43 and FUS influence mitochondrial RNA metabolism in hepatocytes and adipocytes; their aggregation exacerbates mitochondrial dysfunction and oxidative stress, closing the loop with other ageing hallmarks [6]. In cancer, an archetypal age-related disease, RBPs such as HuR, SRSF1, and hnRNPA1 promote oncogenic splicing programmes, stabilise mRNAs encoding cyclins and growth factors, and support the SASP in the tumour microenvironment, thereby linking ageing, senescence, and malignancy [2,29].

In summary, RBPs contribute to organ-specific manifestations of ageing through convergent yet tissue-tailored mechanisms. In the nervous system and muscle, aggregation-prone RBPs such as TDP-43 and FUS predominate [11,13,35]. In the vasculature and heart, HuR and other splicing factors contribute to inflammation and remodelling in a cell-specific manner [38,39]. In metabolic tissues, shifts in TTR-RBPs and core splicing factors promote insulin resistance, steatosis, and senescence [8,10]. Across all systems, these changes amplify SASP-driven sterile inflammation and intersect with proteostasis collapse and mitochondrial dysfunction [1,2]. This tissue heterogeneity highlights both challenges and opportunities for precision gerotherapeutics.

Taken together, evidence from neurodegenerative, musculoskeletal, cardiovascular, and metabolic diseases indicates that RBP dysfunction represents a shared molecular feature across multiple age-related pathologies. However, substantial tissue specificity and context dependency remain major challenges for mechanistic interpretation and therapeutic targeting.

Key takeaway: Although disease manifestations are tissue-specific, RNA-binding protein dysfunction consistently converges on inflammatory amplification, impaired proteostasis, and transcriptomic destabilisation.

## 6. Molecular Mechanisms Underlying RNA-Binding Protein Dysfunction in Ageing and Age-Related Disease

The preceding analysis of organ-specific manifestations has demonstrated that RNA-binding proteins (RBPs) contribute to age-related pathology in a highly context-dependent manner, with TDP-43 and FUS predominating in neurodegenerative and muscle disease, HuR and tristetraprolin (TTP) driving vascular and metabolic inflammation, and core splicing factors such as PTBP1 and heterogeneous nuclear ribonucleoproteins (hnRNPs) promoting senescence-associated isoform switches across tissues [2,10,13]. These phenotypic outcomes originate from a limited set of molecular mechanisms that become progressively dysregulated with advancing age. This section examines these mechanisms in depth: altered RBP expression, post-translational modifications (PTMs), defects in nucleocytoplasmic transport, disruption of liquid–liquid phase separation (LLPS) and pathological aggregation, altered interactions with non-coding RNAs, epitranscriptomic changes, and the establishment of self-reinforcing feedback loops with cellular senescence and other ageing hallmarks [1,2,6].

The most fundamental mechanism is the age-dependent alteration in RBP abundance. Masuda et al. [8] conducted one of the earliest systematic surveys of turnover and translation regulatory RBPs (TTR-RBPs) across 12 human tissues obtained from donors ranging from 0 to 85 years. They reported that HuR (ELAVL1), AUF1 (HNRNPD), and TIA-1 generally declined with donor age in most tissues, whereas TTP (ZFP36) increased. These changes were not uniform; brain and muscle showed distinct patterns compared with liver or kidney. Subsequent studies have confirmed that many core splicing RBPs, including PTBP1, hnRNPUL1, SRSF1, SRSF3, and SRSF7, are consistently downregulated in replicative senescence, progeroid syndromes, and chronologically aged human and murine tissues [2,10]. This downregulation is driven by a combination of transcriptional repression, altered mRNA stability of the RBP transcripts themselves, and increased proteasomal or autophagic degradation. The resulting loss of splicing fidelity produces hundreds of aberrant isoforms that reinforce senescence, mitochondrial dysfunction, and genomic instability, illustrating how changes in RBP levels can amplify multiple ageing hallmarks simultaneously [1,10].

Superimposed on expression changes are extensive post-translational modifications that modulate RBP localisation, RNA-binding affinity, protein–protein interactions, and phase behaviour. Phosphorylation is the best-characterised modification. Stress-activated kinases such as p38 MAPK, JNK, and CK1, whose basal activity rises with age-associated chronic inflammation and oxidative stress, phosphorylate HuR at specific serine residues, promoting its cytoplasmic translocation and enhancing stabilisation of SASP mRNAs (IL-6, IL-8, TNF-α) [2]. Conversely, phosphorylation of TTP by MK2 sequesters it into inactive complexes, removing its destabilising activity and further tilting the balance toward SASP amplification [7]. TDP-43 is hyperphosphorylated at multiple C-terminal serines in both familial and sporadic disease as well as in limbic-predominant age-related TDP-43 encephalopathy (LATE), reducing its RNA-binding capacity and promoting cytoplasmic aggregation [13]. Arginine methylation by PRMT1 and PRMT5 regulates nucleocytoplasmic shuttling of FUS and hnRNPA1; age-related decline in methyltransferase activity or competition for methyl donors impairs nuclear import [6]. Acetylation, ubiquitylation, and PARylation also accumulate with age due to declining NAD+ levels and sirtuin activity, further disrupting RBP function. These PTMs therefore convert acute adaptive responses into chronic maladaptive states characteristic of the aged cellular environment [1,2].

One of the most striking age-related defects involves nucleocytoplasmic transport. Nuclear pore complexes (NPCs) deteriorate with age through oxidative damage, reduced expression of nucleoporins, and disruption of the Ran GTPase gradient. This leads to impaired nuclear import of RBPs that rely on nuclear localisation signals, most notably TDP-43, FUS, and several hnRNPs [6]. The resulting nuclear depletion causes loss-of-function phenotypes, particularly widespread splicing defects in long neuronal pre-mRNAs that contain cryptic exons. At the same time, defective export allows aberrant cytoplasmic accumulation of these proteins, promoting toxic aggregation. Studies in post-mitotic neurons and aged human brain tissue have shown that NPC dysfunction precedes overt proteinopathy and correlates with cognitive decline in both Alzheimer’s disease and LATE [13]. Similar transport defects occur in skeletal muscle and cardiomyocytes, linking NPC ageing to sarcopenia and heart failure. Pharmacological stabilisation of the Ran gradient or NPC integrity has shown promise in delaying RBP mislocalisation in model organisms [2].

Arguably, the most intensively studied mechanism in recent years is the dysregulation of liquid–liquid phase separation and the transition to pathological aggregates. Many ageing-relevant RBPs, including TDP-43, FUS, hnRNPA1, hnRNPA2B1, and TIA-1, contain prion-like low-complexity domains (LCDs) rich in glycine, serine, and glutamine. These domains enable multivalent interactions that drive dynamic, reversible LLPS into stress granules, processing bodies, and other membraneless organelles under acute stress, thereby protecting cells by sequestering mRNAs and translation factors [6]. With advancing age, several factors shift the equilibrium toward irreversible fibrillisation: declining proteostasis capacity (reduced autophagy and proteasome activity), chronic oxidative stress that modifies LCD residues, persistent low-grade inflammation that increases RNA availability for seeding, and PTMs that alter charge distribution within the LCDs [2]. The resulting solid-like aggregates exhibit both loss-of-function (depletion of nuclear RBPs) and toxic gain-of-function (sequestration of other RBPs, mRNAs, and proteins, disruption of nucleocytoplasmic transport, and activation of innate immune pathways via cGAS-STING) [6,11]. In LATE and sporadic ALS/FTD, these aggregates are primarily composed of wild-type rather than mutant protein, emphasising that ageing itself creates a permissive environment for pathology [13].

Crosstalk with non-coding RNAs adds another layer of complexity. Circular RNAs (circRNAs) and certain long non-coding RNAs (lncRNAs) accumulate with age due to their high stability and resistance to exonuclease degradation. Many circRNAs contain multiple RBP-binding sites and function as molecular sponges that titrate HuR, PTBP1, or hnRNPA1 away from their canonical mRNA targets, thereby altering splicing and stability programmes [2]. The lncRNA MALAT1, for example, sequesters SR proteins in nuclear speckles and modulates their phosphorylation, influencing global splicing patterns in senescent cells. Conversely, RBPs regulate the biogenesis, stability, and function of microRNAs and lncRNAs, creating extensive bidirectional regulatory networks that become rewired during ageing. These interactions are particularly prominent in the SASP, where specific lncRNAs stabilised by HuR amplify inflammatory signalling ([15] as cited in [2]).

Emerging evidence also implicates epitranscriptomic modifications. The m6A (N6-methyladenosine) writer, reader, and eraser machinery interact extensively with RBPs. Age-related changes in m6A levels on specific transcripts alter their recognition by YTHDF readers or hnRNPs, affecting stability, splicing, and translation. Oxidative damage to RNA itself, which increases with mitochondrial dysfunction, further modifies RBP binding preferences and promotes erroneous translation [1]. These layers of regulation create highly interconnected networks in which perturbation at one node rapidly spreads throughout the system.

Finally, these molecular mechanisms establish powerful positive-feedback loops with cellular senescence. Downregulation of PTBP1 or hnRNPUL1 induces senescence, which in turn further suppresses splicing factor expression through SASP-mediated paracrine signalling [10]. Cytoplasmic TDP-43 aggregates activate the cGAS-STING pathway, reinforcing SASP and inflammaging, which increases kinase activity that further phosphorylates and mislocalises RBPs [2,13]. Declining proteostasis promotes RBP aggregation, which impairs autophagy, further compromising proteostasis. These self-reinforcing circuits explain why RBP dysfunction accelerates exponentially in later life and why interventions targeting any single node (senolytics, autophagy enhancers, or nuclear transport stabilisers) can produce broad beneficial effects [1].

In summary, RBP dysfunction in ageing arises from the convergence of altered expression, aberrant PTMs, nucleocytoplasmic transport failure, pathological phase transitions, rewired non-coding RNA networks, and epitranscriptomic dysregulation. These mechanisms are not isolated but form an integrated web that links and amplifies the twelve hallmarks of ageing [1]. As Varesi et al. [2] convincingly argue, RBPs may represent a molecular “common linker” across age-related diseases precisely because their dysfunction integrates so many upstream stressors into coherent downstream programmes of senescence and inflammation. A detailed mechanistic understanding of these processes provides the necessary foundation for the development of diagnostic biomarkers and targeted therapeutic interventions, which will be examined in the subsequent section.

**Figure 2:**
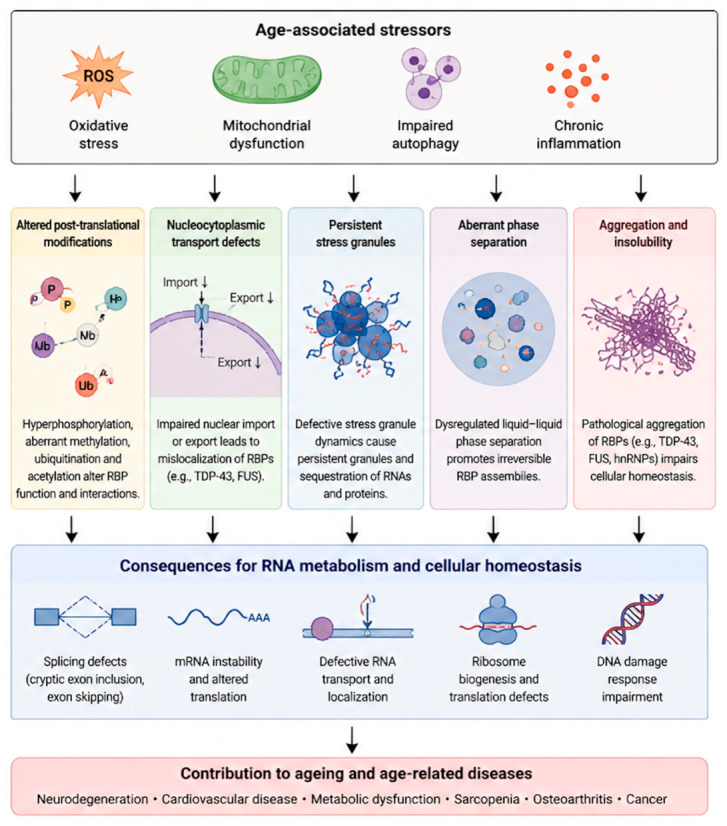
Mechanisms of RNA-Binding Protein Dysfunction During Ageing. Note. Overview of the principal mechanistic pathways through which ageing-associated stress promotes RBP dysfunction and downstream disruption of RNA homeostasis. Created by the authors on 27 May 2026. Synthesised from mechanisms reviewed in [1,2,6,10].

The molecular mechanisms underlying RBP dysfunction during ageing are highly interconnected and involve alterations in post-translational modification, nucleocytoplasmic transport, stress-granule dynamics, phase separation, and proteostasis. Figure 2 provides an overview of the principal mechanistic pathways through which ageing-associated stress promotes RBP dysfunction and downstream disruption of RNA homeostasis.

Overall, age-associated disruption of RNA-binding proteins emerges not as a single isolated process but as a multifactorial collapse of RNA homeostasis involving altered phase separation, impaired nucleocytoplasmic transport, defective stress responses, and declining proteostatic capacity. These interconnected mechanisms likely reinforce one another during biological ageing.

Key takeaway: The convergence of RNA metabolic dysfunction across ageing hallmarks establishes RNA-binding proteins as promising biomarkers and therapeutic targets for precision geroscience.

## 7. Biomarker Potential and Therapeutic Targeting of RNA-Binding Proteins in Ageing and Age-Related Disease

The detailed molecular mechanisms described in the previous section (including altered expression, aberrant post-translational modifications, nucleocytoplasmic transport defects, pathological liquid–liquid phase separation, disrupted interactions with non-coding RNAs, and self-reinforcing feedback loops with senescence) provide a robust framework for both diagnostic biomarker development and therapeutic intervention [1,2,6]. Because RBPs sit at the convergence point of multiple ageing hallmarks and act as common molecular linkers across diverse age-related diseases, they offer unique opportunities for biomarkers that reflect integrated biological age rather than single-pathway disruption, as well as for therapies that could simultaneously ameliorate senescence, chronic inflammation, proteostasis collapse, and mitochondrial dysfunction [2,10]. This final substantive section evaluates the current state and future potential of RBP-based biomarkers and therapeutic strategies, drawing exclusively on peer-reviewed evidence while acknowledging the substantial translational challenges that remain.

### 7.1. Biomarker Potential

Several classes of RBP-related biomarkers have shown promise in human studies. First, circulating levels or post-translationally modified forms of RBPs can be detected in plasma, serum, or extracellular vesicles. TDP-43 pathology, for example, is detectable as elevated phosphorylated TDP-43 or truncated TDP-43 fragments in cerebrospinal fluid and, more recently, in blood-based assays. In individuals with limbic-predominant age-related TDP-43 encephalopathy (LATE), plasma TDP-43 levels and their seeding activity correlate with cognitive decline and hippocampal atrophy independently of Alzheimer’s disease biomarkers [13,14]. Similarly, elevated extracellular vesicle-associated TDP-43 or FUS has been reported in amyotrophic lateral sclerosis (ALS), frontotemporal dementia (FTD), and even in a subset of cognitively normal older adults, suggesting utility for early detection of age-related proteinopathy [6].

HuR and other turnover and translation regulatory RBPs (TTR-RBPs) also show biomarker potential. Cytoplasmic HuR levels in peripheral blood mononuclear cells or plasma exosomes correlate with frailty indices, systemic inflammatory markers, and sarcopenia severity in older adults [8,29]. The ratio of HuR to tristetraprolin (TTP/ZFP36) activity, measured indirectly through the stability of shared target mRNAs such as IL-6 or TNF-α transcripts in circulating immune cells, has been proposed as an inflammaging index. Reduced levels of splicing factors such as PTBP1 or SRSF1 in plasma or muscle biopsies correlate with senescent cell burden and predict physical performance decline in longitudinal ageing cohorts [2,10].

A second promising biomarker category involves RBP-regulated RNA isoforms or alternative splicing signatures. Because downregulation of PTBP1, hnRNPUL1, and related splicing RBPs produces a consistent senescence-associated splicing signature across multiple tissues, panels of differentially spliced exons (for example, in genes regulating DNA repair, autophagy, or mitochondrial function) can serve as robust molecular clocks of biological age [10]. Long-read RNA sequencing of blood or peripheral tissues has revealed that intron retention events and cryptic exon inclusion, both hallmarks of nuclear TDP-43 loss, increase with chronological age and are accelerated in neurodegenerative disease [2,12]. These splicing biomarkers may offer greater specificity than traditional inflammatory markers because they reflect upstream RBP dysfunction rather than downstream consequences.

Third, epitranscriptomic and phase-separation-related readouts are emerging. Changes in m6A-modified RNAs bound by specific RBPs (such as YTHDF2 or hnRNPs) can be profiled in circulating RNA, while the abundance of certain circular RNAs that sponge HuR or PTBP1 correlates with cognitive frailty and cardiovascular risk in older populations ([15] as cited in [2]). Finally, functional biomarkers that integrate RBP activity, such as the cytoplasmic-to-nuclear ratio of HuR or TDP-43 measured by imaging flow cytometry in immune cells, may provide dynamic readouts of therapeutic response.

These biomarker candidates are advantageous because they are mechanistically anchored in the core biology of ageing rather than being mere correlates of disease. However, challenges remain, including tissue specificity, the influence of comorbidities, and the need for standardised assays. Large-scale longitudinal studies integrating multi-omics with clinical outcomes will be required to validate RBP-based composite ageing clocks [1,2].

### 7.2. Therapeutic Targeting of RBPs

The druggability of RBPs has improved substantially in the past decade, moving from what was once considered “undruggable” to a rapidly expanding therapeutic modality. Strategies can be grouped into four broad categories: restoration of physiological RBP levels or localisation, modulation of RBP–RNA interactions, prevention or reversal of pathological aggregation and phase separation, and indirect targeting through senescence or proteostasis pathways.

Direct restoration approaches include antisense oligonucleotides (ASOs) and small interfering RNAs designed to correct splicing defects caused by nuclear depletion of TDP-43 or downregulation of PTBP1. ASOs that promote inclusion of protective exons or exclusion of cryptic exons have already shown success in spinal muscular atrophy and are being adapted for TDP-43-related cryptic exon repression in ALS and LATE models [6]. Viral vector-mediated delivery of splicing factors such as PTBP1 or specific SRSF isoforms has delayed senescence in cultured cells and improved muscle function in aged mice, although systemic delivery and off-target effects require careful optimisation [2,10].

Small-molecule modulators of RBP activity represent another active area. HuR inhibitors that block its RNA-binding pocket or prevent cytoplasmic translocation have demonstrated efficacy in reducing SASP output and inflammation in models of osteoarthritis, atherosclerosis, and cancer-associated senescence [29]. Compounds that stabilise TTP activity or enhance AUF1 function could restore balance to AU-rich element mRNA metabolism and dampen chronic inflammation [7,8]. For aggregation-prone RBPs, small molecules that stabilise the native conformation of TDP-43 or FUS, or that inhibit deleterious phase transitions, are in preclinical development. These include compounds that modulate the prion-like domain or enhance nuclear import by stabilising the Ran gradient [6].

Autophagy-enhancing and senolytic therapies indirectly benefit RBP homeostasis. Clearance of senescent cells with dasatinib plus quercetin or navitoclax partially restores PTBP1, SRSF, and HuR expression profiles in aged tissues and reduces pathological TDP-43 aggregation [2]. mTOR inhibitors and NAD+ precursors improve proteostasis, thereby reducing RBP aggregation and normalising phase separation dynamics. Nuclear transport modulators that restore nucleoporin function or Ran GTPase activity have shown neuroprotective effects in TDP-43 and FUS models [13].

Emerging RNA-targeted therapeutics, including small molecules that bind specific RNA structures to block aberrant RBP binding and CRISPR-based approaches to edit RBP-binding sites, offer high specificity. Oligonucleotide therapies that degrade toxic circRNAs or lncRNAs that sequester beneficial RBPs are also under investigation [2]. Because many RBPs regulate overlapping but non-identical transcript networks, combination therapies that simultaneously target HuR inhibition, splicing factor restoration, and autophagy enhancement may produce synergistic effects on healthspan.

Proof-of-concept studies support translational potential. In C. elegans and Drosophila, genetic modulation of orthologues of TDP-43, FUS, or PTBP1 extends lifespan and improves proteostasis. In mice, muscle-specific HuR deletion or systemic low-dose HuR inhibitors ameliorates sarcopenia and metabolic dysfunction, while ASO-mediated correction of TDP-43-dependent splicing improves motor function in ALS models [6]. Early-phase human trials of splicing modulators and HuR-targeted agents in cancer and inflammatory diseases provide safety data that can be repurposed for ageing indications.

### 7.3. Challenges, Limitations, and Future Directions

Despite the compelling mechanistic insights and translational promise outlined throughout this review, several important limitations must be acknowledged. First, the majority of cellular mechanistic data derive from replicative senescence models using fetal or young-adult fibroblasts subjected to serial passaging, oncogene activation, or DNA-damage agents. These models capture certain aspects of cellular ageing but fail to fully recapitulate the complex, multi-factorial, and chronic nature of organismal ageing in vivo, including the influence of systemic inflammation, changing hormonal milieu, and tissue-specific microenvironments [1,2]. Consequently, it remains difficult to determine whether observed RBP changes are causal drivers or downstream correlates of senescence. Although experimental depletion of PTBP1 or hnRNPUL1 induces senescence-like phenotypes [10], and clearance of senescent cells partially restores RBP profiles, truly longitudinal causal studies in naturally aged mammals are still scarce. Future studies should prioritise longitudinal, tissue-specific genetic models and single-cell multi-omics approaches to better distinguish cause from consequence. In parallel, low-cost and scalable interventions such as social prescribing deserve greater attention in future research. Ferreira et al. [40] demonstrated that social prescribing programmes significantly improved self-esteem in older adults (*p* < 0.001), with a positive trend in cognitive performance, reinforcing the value of holistic approaches that target the social determinants of ageing and may complement molecular strategies directed at RBP-mediated post-transcriptional regulation. These biological insights should be integrated with psychological and existential perspectives on ageing; as Alves Ferreira [41] argues, boredom can function as a sign of existential disconnection and subjective anomie, while related work examines daily proactivity under adverse social conditions [42], the role of faith and hope in the human condition [43], stratified relational models of consciousness and responsibility [44], and multispecies approaches to planetary health education [45].

Second, substantial tissue, cell-type, and even subcellular heterogeneity exists. The same RBP (for example, HuR) can be protective in vascular smooth muscle cells yet deleterious in endothelial cells or macrophages [38]. Similarly, TDP-43 loss-of-function in neurons produces distinct splicing defects compared with its effects in glia or myocytes. Most studies examine only one or two cell types, limiting generalisability. Single-cell and spatial transcriptomic approaches are beginning to address this gap, but integration with RBP-binding maps (CLIP-seq) across the human lifespan remains technically challenging and costly.

Third, distinguishing loss-of-function from gain-of-toxic-function in prion-like RBPs remains contentious. Nuclear depletion of TDP-43 clearly produces splicing defects, yet cytoplasmic aggregates may exert independent toxicity through sequestration of other RBPs and activation of innate immunity [6,13]. The relative contribution of each mechanism likely varies by disease stage, genetic background, and environmental exposures, complicating therapeutic design. Furthermore, most aggregation studies use overexpression or mutant proteins; whether findings translate to the more subtle, progressive mislocalisation of wild-type protein in normative ageing is not fully established.

Fourth, methodological limitations in biomarker development are notable. Circulating TDP-43 and HuR measurements are influenced by haemolysis, comorbidities, and acute inflammatory states, reducing specificity. Splicing signatures, while promising, require long-read sequencing that is not yet routine in clinical laboratories. Standardisation of pre-analytical variables and establishment of age- and sex-specific reference ranges will be essential before these can enter clinical practice.

Fifth, therapeutic targeting faces substantial hurdles. Systemic modulation of pleiotropic RBPs such as HuR or PTBP1 risks on-target toxicity in tissues where their activity is beneficial. Delivery across the blood–brain barrier for neurodegenerative indications remains challenging. Off-target effects on non-coding RNA networks and epitranscriptomic regulation are difficult to predict and monitor. Moreover, most preclinical efficacy data derive from short-term interventions in disease models rather than lifelong prevention studies in naturally aged animals. Long-term safety, particularly regarding cancer risk given the tumour-suppressive functions of senescence, requires careful evaluation.

Finally, conceptual limitations persist. The field has yet to establish a unified quantitative framework that integrates RBP expression, binding dynamics, phase behaviour, and downstream RNA outputs into a predictive model of biological age. Interdisciplinary collaboration between RNA biologists, geroscientists, clinicians, and bioinformaticians will be necessary to move from descriptive catalogues of changes to causal network models and personalised interventions.

Emerging transcriptomic and spatial transcriptomic approaches are beginning to reveal tissue-specific patterns of RBP dysregulation during ageing, including age-associated disruption of RNA-processing networks across distinct cellular populations [46].

These limitations do not diminish the importance of RBPs in ageing biology but rather highlight priority areas for future research: development of more physiologically relevant models, single-cell multi-omics integration, rigorous causal validation using inducible and cell-type-specific genetic tools, and biomarker-driven early-phase clinical trials. Addressing these gaps will be essential to translate the substantial mechanistic knowledge accumulated over the past two decades into meaningful clinical advances that extend human healthspan.

Given the profound tissue specificity and context dependency of RBP function, future therapeutic strategies will likely require precision-targeted rather than systemic approaches. Potential strategies may include antisense oligonucleotides, RNA-targeted small molecules, cell-specific nanoparticle delivery systems, splice-switching therapeutics, and modulation of stress-granule dynamics. Importantly, therapies aimed at restoring physiological RBP localisation or phase behaviour may prove safer than complete inhibition of multifunctional RBPs with essential housekeeping roles.

#### 7.3.1. Challenges in Establishing Causality

A major limitation across the current literature on RNA-binding proteins (RBPs) and ageing is the predominance of correlational evidence. Although numerous studies document age-associated alterations in RBP abundance, localisation, post-translational modification, phase-separation behaviour, and RNA-binding activity, relatively few studies conclusively demonstrate whether these alterations are primary causal drivers of ageing or secondary consequences of accumulated cellular damage.

Most mechanistic evidence derives from in vitro knockdown or overexpression systems in fibroblasts or immortalised cell lines. While depletion of RBPs such as PTBP1, hnRNPUL1, and several serine/arginine-rich splicing factors can induce senescence-associated phenotypes [10], the physiological relevance of these findings to natural mammalian ageing remains incompletely established. Conditional, tissue-specific, and temporally controlled in vivo models are still comparatively limited.

Similarly, aggregation-prone RBPs such as TDP-43 and FUS exhibit both toxic gain-of-function and loss-of-function properties, complicating causal interpretation. Cytoplasmic aggregation may reflect impaired proteostasis and defective autophagy rather than representing a primary initiating event. Distinguishing initiating pathogenic mechanisms from downstream stress responses therefore remains a major unresolved challenge.

An additional complexity arises from profound tissue and cell-type heterogeneity. The same RBP may exert protective functions in one cellular context while contributing to pathology in another. HuR, for example, displays pro-inflammatory functions in endothelial cells and macrophages yet may exert protective effects in vascular smooth muscle cells [38,39]. Likewise, nuclear TDP-43 is essential for normal RNA processing, whereas cytoplasmic aggregation is neurotoxic. These context-dependent properties complicate therapeutic targeting and increase the risk of off-target effects.

Future progress will require longitudinal ageing studies, inducible rescue paradigms in aged animals, single-cell multi-omics, spatial transcriptomics, and tissue-specific perturbation systems capable of resolving dynamic RBP behaviour across the lifespan. Such approaches will be essential to determine whether modulation of RBPs can genuinely alter biological ageing trajectories rather than merely modifying downstream manifestations of tissue dysfunction.

Emerging single-cell transcriptomic, spatial transcriptomic, and epitranscriptomic technologies are likely to transform understanding of RBP biology during ageing. These approaches now permit cell-type-specific mapping of splicing alterations, stress-granule dynamics, and RNA-processing defects within intact tissues, overcoming many limitations of bulk transcriptomic analyses. Integration of multi-omics datasets with longitudinal ageing models may ultimately allow identification of tissue-specific RBP signatures predictive of biological ageing trajectories and therapeutic responsiveness.

Despite the growing body of evidence linking RBPs to ageing and age-related disease, the strength of causal evidence varies substantially across proteins and experimental systems. Table 2 summarises the current balance between correlational and mechanistic evidence for major RBPs implicated in ageing biology.

**Figure 3:**
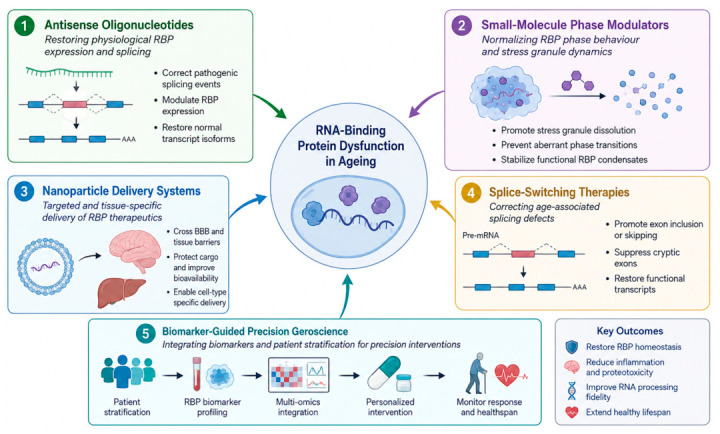
Translational roadmap for therapeutic targeting of RNA-binding proteins in ageing. Note. The schematic summarises major intervention strategies targeting age-associated RNA-binding protein dysfunction, including antisense oligonucleotide-based correction, small-molecule phase-separation modulators, nanoparticle-mediated delivery systems, splice-switching therapeutics, and biomarker-guided precision geroscience approaches. Created by the authors on 27 May 2026. Synthesised from mechanisms reviewed in [1,2,6,10].

To bridge mechanistic insight with translational application, Figure 3 summarises emerging therapeutic strategies aimed at restoring RNA-binding protein homeostasis across ageing-associated pathologies.

#### 7.3.2. Critical Perspective and a Path Forward

Despite the rapid expansion of research linking RNA-binding proteins (RBPs) to ageing and age-related disease, the field remains conceptually fragmented and methodologically heterogeneous. Many studies focus on isolated RBPs, single tissues, or specific disease contexts, making it difficult to determine whether RBP dysregulation represents a unifying driver of biological ageing or merely a downstream consequence of cellular stress and degeneration.

A major conceptual challenge is that RBPs rarely function in isolation. Instead, they operate within highly interconnected post-transcriptional regulatory networks involving non-coding RNAs, stress granules, spliceosomal complexes, epitranscriptomic modifications, and proteostasis pathways. Consequently, alterations observed in one RBP may reflect broader systemic collapse of RNA homeostasis rather than protein-specific pathogenicity.

Furthermore, the same RBP may exert protective or deleterious effects depending on cellular context, tissue type, subcellular localisation, and disease stage. HuR, for example, can promote inflammatory signalling in endothelial cells and macrophages while simultaneously exerting stabilising and reparative functions in vascular smooth muscle cells [38,39]. Likewise, nuclear TDP-43 is essential for normal neuronal RNA processing, whereas cytoplasmic TDP-43 aggregation is strongly neurotoxic. These dual roles complicate simplistic therapeutic paradigms based solely on inhibition or activation of individual RBPs.

Collectively, these considerations suggest that future progress in the field will require systems-level approaches integrating transcriptomics, proteomics, spatial biology, and longitudinal ageing models. Rather than viewing RBPs as isolated molecular actors, emerging evidence supports their interpretation as dynamic regulators of RNA homeostasis operating across multiple hallmarks of ageing simultaneously. A viable path forward includes the use of inducible, cell-type-specific knockout models in aged animals, combined with single-cell multi-omics and spatial transcriptomics to resolve context-dependent RBP functions. Only then can we design interventions that selectively target the pathogenic aspects of RBP dysregulation without disrupting their essential physiological roles.

Taken together, these challenges highlight the need to transition from descriptive and reductionist approaches toward integrative, systems-level frameworks capable of capturing the dynamic and context-dependent nature of RBP function across the lifespan.

### 7.4. Limitations of the Review

This review has several limitations inherent to its narrative design. The selection of studies was not conducted through a systematic or meta-analytic framework, which may introduce selection bias. Although emphasis was placed on the high-quality and recent literature, some relevant studies may not have been included.

Additionally, the interpretation of findings is dependent on the heterogeneity of the underlying literature, including variability in experimental models, analytical approaches, and definitions of ageing-related phenotypes.

Finally, given the rapidly evolving nature of the field, particularly with advances in single-cell and spatial transcriptomics, some concepts discussed here may require refinement as new data emerge (see Section 9. Limitations and Gaps in the Current Literature).

## 8. Discussion

The preceding sections collectively demonstrate that RNA-binding proteins occupy a uniquely privileged position in the biology of ageing. Far from being passive housekeeping factors, RBPs actively integrate and amplify the twelve hallmarks articulated by López-Otín et al. [1]. The systematic downregulation of core splicing regulators such as PTBP1 and hnRNPUL1 generates a coherent senescence-associated splicing programme that reinforces cell-cycle arrest, impairs autophagy and DNA repair, and disrupts mitochondrial homeostasis [10]. Concurrently, the rebalancing of TTR-RBPs (increased functional dominance of HuR and functional inactivation of TTP and specific AUF1 isoforms) converts transient inflammatory responses into the persistent SASP that drives inflammaging and paracrine senescence propagation [2,8]. In parallel, the prion-like RBPs TDP-43, FUS, and hnRNPA1 undergo progressive nuclear depletion and cytoplasmic aggregation, linking proteostasis failure directly to splicing defects, impaired local translation, and activation of innate immune pathways [6,11,13].

This integrative capacity explains why RBPs can be conceptualised as “common linkers” across seemingly disparate age-related diseases [2]. The same molecular machinery that supports adaptive stress responses in youth becomes maladaptive in the aged cellular environment characterised by declining proteostasis, chronic low-grade inflammation, and impaired nucleocytoplasmic transport. The resulting self-reinforcing loops (whereby RBP dysfunction induces senescence, which further suppresses splicing factor expression and increases kinase activity that mislocalises additional RBPs) create a molecular “vicious cycle” that accelerates decline after a certain threshold. This dynamic is consistent with the antagonistic pleiotropy theory of ageing: mechanisms that are beneficial for fitness early in life (rapid stress granule formation, tight control of inflammatory transcripts, efficient alternative splicing) become detrimental when chronically engaged later in life.

The tissue-specific manifestations reviewed here further enrich this picture. In the brain, post-mitotic neurons with extreme polarisation and high metabolic demand are particularly vulnerable to disruptions in mRNA transport and local synaptic translation, explaining the prominence of TDP-43 and FUS pathology in ALS, FTD, and LATE [13]. In skeletal muscle, the convergence of neurogenic (TDP-43) and myogenic (HuR, PTBP1) RBP defects provides a molecular explanation for the intimate link between motor neuron ageing and sarcopenia [35]. In the vasculature, the opposing effects of endothelial versus smooth-muscle HuR illustrate the necessity of cell-type-specific targeting strategies [38]. These observations reinforce the view that successful gerotherapeutics will likely require precision delivery or biomarker-guided patient stratification rather than systemic, non-specific modulation.

From a geroscience perspective, the therapeutic tractability of RBPs is particularly encouraging. Unlike many transcription factors, RBPs can be targeted by small molecules that modulate RNA-binding pockets, phase-separation propensity, or specific protein–RNA interfaces. Antisense oligonucleotides have already proven clinically successful for splicing modulation in neuromuscular disease and are being adapted for TDP-43-related cryptic exon repression. Senolytic and autophagy-enhancing strategies indirectly restore RBP homeostasis, suggesting that combination therapies may yield synergistic benefits across multiple hallmarks [1,2]. The development of composite biomarkers (incorporating circulating phosphorylated TDP-43, cytoplasmic HuR ratios, and specific splicing signatures) could enable early detection of accelerated biological ageing before overt clinical disease manifests.

Critically, the literature reveals both convergence and divergence. While downregulation of splicing factors and aggregation of prion-like RBPs appear near-universal features of ageing, the directionality and functional consequences of TTR-RBP changes are highly context-dependent. This heterogeneity underscores the need for systems-level network analyses that integrate RBP-CLIP data, long-read transcriptomics, and single-cell proteomics across the lifespan. Such approaches will be essential to distinguish driver from passenger events and to identify the highest-leverage nodes for intervention.

In conclusion, the collective evidence synthesised in this review establishes RBPs as central, actionable regulators of the ageing process. Their position at the intersection of RNA metabolism, phase separation biology, and senescence positions them as high-value targets for next-generation gerotherapeutics. Realising this potential will require overcoming the methodological and conceptual limitations discussed below, yet the substantial mechanistic foundation already established justifies cautious optimism that modulation of RNA-binding proteins may ultimately contribute to compressing morbidity and extending healthy human lifespan.

## 9. Limitations and Gaps in the Current Literature

Several important limitations currently constrain the interpretation and translational applicability of the RBP ageing literature.

First, much of the existing evidence remains heavily correlational. Numerous studies demonstrate age-associated alterations in RBP abundance, localisation, aggregation, or splicing activity, yet relatively few establish direct causality in naturally aged mammalian systems. Many mechanistic experiments rely on acute knockdown or overexpression paradigms in immortalised cell lines, which may not accurately recapitulate the complexity of organismal ageing.

Second, substantial tissue and cell-type heterogeneity complicates interpretation. The same RBP may exert opposing functions depending on cellular context, disease stage, or subcellular localisation. Consequently, findings derived from isolated tissues or simplified cellular systems may not generalise across organ systems.

Third, the field remains methodologically fragmented. Different studies employ heterogeneous transcriptomic pipelines, senescence models, ageing definitions, and experimental endpoints, limiting direct comparability between datasets.

Fourth, many studies focus predominantly on neurodegenerative disease, particularly TDP-43 and FUS proteinopathies, whereas the roles of RBPs in cardiovascular ageing, metabolic dysfunction, immune ageing, and musculoskeletal decline remain comparatively underexplored.

Finally, human longitudinal data remain limited. Most human studies rely on post-mortem tissue analyses or cross-sectional comparisons, making it difficult to determine temporal relationships between RBP dysfunction and disease progression. This absence of longitudinal causal evidence remains the single greatest barrier to translating RBP biology into effective gerotherapies.

Addressing these limitations will require a shift toward longitudinal, multi-omics, and systems-level approaches, integrating single-cell and spatial transcriptomics with functional validation in physiologically relevant ageing models. Such advances will be essential to move from descriptive associations to predictive and mechanistically grounded frameworks of RBP-driven ageing.

## 10. Conclusions

RNA-binding proteins (RBPs) have emerged as central regulators of RNA homeostasis whose dysfunction intersects with multiple hallmarks of biological ageing. Beyond their canonical roles in mRNA splicing, stability, localisation, and translation, RBPs increasingly appear to function as systems-level integrators linking senescence, chronic inflammation, proteostasis collapse, mitochondrial dysfunction, and neurodegeneration.

Importantly, the evidence reviewed here suggests that RBP dysfunction is unlikely to represent a single linear pathogenic pathway. Rather, ageing-associated alterations in RBPs appear to arise through complex bidirectional interactions between cellular stress, declining proteostatic capacity, altered phase separation dynamics, and progressive disruption of post-transcriptional regulation. This complexity likely explains the pronounced tissue specificity and context dependency observed across age-related diseases.

Although significant mechanistic and translational challenges remain, emerging technologies including single-cell transcriptomics, spatial omics, epitranscriptomics, and inducible ageing models are beginning to provide the resolution necessary to dissect these dynamic RNA-regulatory networks in vivo. Future integration of these approaches may ultimately determine whether modulation of RBPs can meaningfully alter biological ageing trajectories or primarily mitigate downstream pathological consequences.

Collectively, current evidence supports the view that RBPs represent not merely passive markers of ageing but potentially fundamental regulators of age-associated cellular resilience and vulnerability. Understanding how RNA homeostasis deteriorates across the lifespan may therefore provide new opportunities for biomarker development and therapeutic intervention across multiple age-related diseases.

## Figures and Tables

**Table 1:** Key RNA-Binding Proteins Implicated in Ageing and Age-Related Disease.

RBP	Main Functions	Age-Related Changes	Associated Diseases	Therapeutic Potential
HuR (ELAVL1)	mRNA stabilisation, stress response, SASP regulation	Increased cytoplasmic localisation, altered phosphorylation	Atherosclerosis, sarcopenia, osteoarthritis, neuroinflammation	HuR inhibitors, modulation of cytoplasmic shuttling
AUF1 (HNRNPD)	ARE-mediated mRNA decay	Decline of protective isoforms	Chronic inflammation, metabolic dysfunction	Isoform-specific restoration
TTP (ZFP36)	Destabilisation of inflammatory mRNAs	Functional inactivation via phosphorylation	Chronic inflammation, cardiovascular disease	TTP activation, MK2 inhibition
PTBP1	Alternative splicing regulation	Downregulation during senescence	Senescence, neurodegeneration	Splicing correction therapies
hnRNPUL1	DNA repair, splicing regulation	Reduced expression	Senescence, genomic instability	RNA-based therapies
SRSF1/SRSF7	Splicing fidelity	Reduced expression/activity	Progeroid phenotypes, cancer, ageing	Small-molecule splice modulators
TDP-43	Splicing, RNA transport	Nuclear depletion, cytoplasmic aggregation	ALS, FTD, LATE, sarcopenia	Antisense oligonucleotides, aggregation inhibitors
FUS	DNA repair, splicing, phase separation	Aberrant phase separation, aggregation	ALS, FTD	Nuclear import restoration
TIA-1	Stress granule nucleation	Persistent stress granules	Neurodegeneration	Modulation of phase separation
G3BP1/G3BP2	Stress granule assembly	Dysregulated stress granule dynamics	Alzheimer’s disease	Stress-granule-targeted therapies
SFPQ	RNA processing, transcriptional regulation	Downregulation in AD tissues	Alzheimer’s disease	Nuclear function restoration
YBX1	Translation and stress-response regulation	Altered expression and localisation	Osteoarthritis, ageing cartilage	Translational modulation

Note: ARE = AU-rich element; SASP = senescence-associated secretory phenotype; ALS = amyotrophic lateral sclerosis; FTD = frontotemporal dementia; LATE = limbic-predominant age-related TDP-43 encephalopathy; AD = Alzheimer’s disease. Therapeutic potential is based on preclinical studies and may not reflect clinical validation.

**Table 2:** Evidence Supporting Correlative Versus Causal Roles of RNA-Binding Proteins in Ageing.

RNA-Binding Protein	Predominant Evidence Type	Key Findings	Strength of Causal Evidence	Main Limitation
PTBP1	siRNA depletion and senescence models	Loss induces senescence-associated splicing defects and mitochondrial dysfunction	Moderate	Mostly derived from in vitro systems
hnRNPUL1	Cellular senescence transcriptomics	Downregulation associated with DNA repair defects and senescence	Weak–Moderate	Limited in vivo ageing evidence
SRSF family proteins	Knockdown studies and splice analysis	Splicing dysregulation contributes to proliferative arrest	Moderate	Lack of longitudinal mammalian studies
HuR (ELAVL1)	Pharmacological and genetic modulation	Alters SASP regulation and inflammatory signalling	Moderate	Strong tissue-specific heterogeneity
TTP (ZFP36)	Knockout mouse models	Loss promotes chronic inflammatory phenotypes	Moderate–Strong	Limited direct lifespan studies
TDP-43	Transgenic animal models and neuropathology	Aggregation and cryptic exon inclusion linked to neurodegeneration	Strong in disease; moderate in ageing	Difficult to separate ageing from neurodegeneration
FUS	Aggregation and phase-separation models	Aberrant condensates disrupt RNA metabolism	Moderate	Limited ageing-specific in vivo evidence
SFPQ/G3BP2	Human AD tissue studies	Downregulation associated with stress-granule dysfunction	Weak	Predominantly correlational human data

## Data Availability

No new data were created or analysed in this study.
